# Covert preparation of a manual response in a ‘go’/‘no-go’ saccadic task is driven by execution of the eye movement and not by visual stimulus occurrence

**DOI:** 10.3389/fnhum.2015.00556

**Published:** 2015-10-02

**Authors:** Claudio Maioli, Luca Falciati

**Affiliations:** Department of Clinical and Experimental Sciences and National Institute of Neuroscience, University of Brescia, BresciaItaly

**Keywords:** visually guided saccades, selective attention, transcranial magnetic stimulation, motor planning, double-choice task

## Abstract

It has been recently demonstrated that visually guided saccades are linked to changes in muscle excitability in the relaxed upper limb, which are compatible with a covert motor plan encoding a hand movement toward the gaze target. In this study we investigated whether these excitability changes are time locked to the visual stimulus, as predicted by influential attention models, or are strictly dependent on saccade execution. Single-pulse transcranial magnetic stimulation was applied to the motor cortex at eight different time delays during a ‘go’/‘no-go’ task, which involved overt or covert orienting of attention. By analyzing the time course of excitability in three hand muscles, synchronized with the onset of either the attentional cue or the eye movement, we demonstrated that side- and muscle-specific excitability changes were strictly time locked to the saccadic response and were not correlated to the onset of the visual attentive stimulus. Furthermore, muscle excitability changes were absent following a covert shift of attention. We conclude that a sub-threshold manual motor plan is automatically activated by the saccade decision-making process, as part of a covert eye-hand coordination program. We found no evidence for a representation of spatial attention within the upper limb motor map.

## Introduction

In everyday life, most common motor tasks are executed under visual guidance, and the hand rarely moves without being coupled to gaze. It has been recently demonstrated that visually guided saccadic eye movements are linked to the excitability modulation of the upper limb cortico-spinal system (CSS; Falciati et al., 2013). These excitability changes are compatible with a covert motor plan encoding an aiming movement of the hand toward the eye target, even if a manual response is not required by the task. This finding can be interpreted in accordance with two alternative explanations, linking the excitability changes of the arm CSS either: (1) to the engagement of spatial attention toward a visual stimulus, or (2) to the actual execution of an overt oculomotor response to a peripheral target.

The first hypothesis is based on the role of visual attention in spatial motor control, proposed by two influential models which posit the occurrence of a strong link between sensory and motor representations of action. According to the “Visual-Attention-Model” of Schneider (1995), the abrupt onset of a salient visual stimulus engages a “selection-for-action” process (Allport, 1987), leading to the simultaneous programming in the dorsal visual stream of possible spatial motor actions (saccades, pointing, reaching, grasping) toward the same target (Schneider and Deubel, 2002; Schiegg et al., 2003). A similar prediction has also been suggested by the “Premotor Theory” of spatial attention (Rizzolatti et al., 1987). According to this model, a shift of spatial attention results from the activation of ‘pragmatic maps’, which correspond to the brain areas engaged in the programming of body movements. Although originally proposed for eye movements, this idea was later generalized to all goal-directed, spatially coded movements (Rizzolatti et al., 1994). The described changes in CSS excitability (Falciati et al., 2013) could then be interpreted as covert programming of a manual goal-directed movement in parallel with the saccade, which is directly elicited by the abrupt appearance of the peripheral cue. According to this viewpoint, the shift of selective attention toward the peripheral cue is determined by the preparation of multiple goal-directed movement with different effectors. Obviously, in both models, the covert activation of multiple motor programs does not necessarily imply overt execution, which, in contrast, requires that a separate ‘go’ control signal be issued, depending on what is dictated by the task.

The second hypothesis, instead, stems from compelling evidence demonstrating a strong dependency of goal-directed hand movements on overt gazing behavior (Mennie et al., 2007). Typically, the eyes start to move toward a target and reach it before the hand (Prablanc et al., 1979; Lünenburger et al., 2000; Sailer et al., 2000; Dean et al., 2011). Furthermore, subjects tend to fixate most of the time the goal of the visually guided manual task (Ballard et al., 1995; Pelz and Canosa, 2001), and spatial reaching errors increase if actors do not look at their target (Vercher et al., 1994; Henriques et al., 1998; Neggers and Bekkering, 1999; van Donkelaar and Staub, 2000; Horstmann and Hoffmann, 2005). Actually, in tasks requiring manual interaction with objects, saccades are not normally directed to the most salient points of the visual scene but rather to locations that are relevant for guiding the ongoing motor act (Land et al., 1999; Hayhoe et al., 2003; Hayhoe and Ballard, 2005). These studies suggest strict coupling of manual motor programming to saccade execution, rather than to the occurrence of a relevant visual stimulus.

Transcranial magnetic stimulation (TMS) is a powerful tool that allows to disclose an ongoing motion planning by dynamically measuring the excitability changes of the corticospinal system (CSS) in behaving subjects, as estimated from motor evoked potential (MEP) amplitude in the relaxed muscles (Schütz-Bosbach et al., 2008). By using TMS, in this study we investigate whether a covert motor program for a hand movement is elicited solely by the presentation of a salient visual stimulus, independent of the final decision to make a gaze shift, or whether the engagement of the hand motor system is strictly coupled to the execution of a saccadic eye movement. To this end, we analyzed the time course of CSS excitability for distal upper limb muscles while subjects executed a ‘go’/‘no-go’ task on a peripheral visual stimulus, randomly imposing either a gaze shift or the maintenance of central fixation.

## Materials and Methods

### Subjects

Twenty adult volunteers (12 males and 8 females, mean age: 21.5 years, range: 19–30) with no history of head trauma or neurological disease participated in the study. All of the subjects were right handed (as measured by the Edinburgh handedness inventory) and naïve to the purpose of the experiment. This study was conducted in accordance with the recommendations of the local Ethics Committee and with the ethical guidelines set forth by the Declaration of Helsinki. Written informed consent was obtained from all participants.

### Experimental Protocol

**FIGURE 1:**
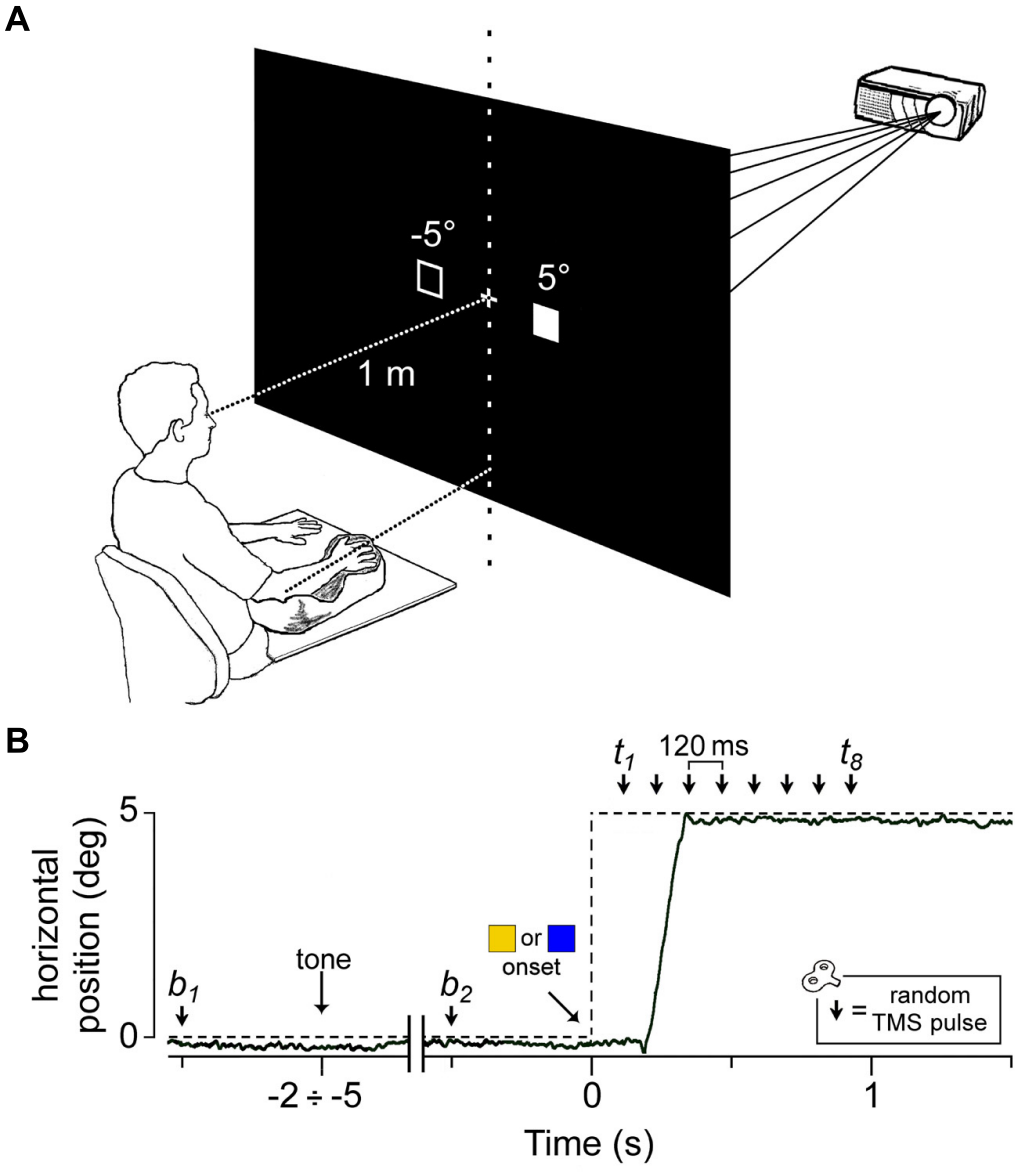
**Experimental setup and protocol. (A)** Visual stimuli were rear-projected on a wide-tangent black screen placed 1 m in front of the subject. The filled square represents the actual location of the ‘go’/’no-go’ cue, while the empty square indicates the other possible position. The head was immobilized using a chin rest and a head-support device (not shown). Notice the arm-hand posture imposed on the subject with respect to the central vertical meridian (see text). **(B)** An electrooculography (EOG) recording (solid line) during a representative saccadic response in a ‘go’ trial. The dashed line indicates the time course of the visual stimulation. The peripheral cue (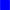 or 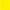) appeared at a variable time interval of 2–5 s after a warning tone. A single pulse of Transcranial magnetic stimulation (TMS) was randomly delivered either before cue onset (at *b*_1_ or *b*_2_) or at one of eight equally spaced time delays between 120 ms and 960 ms (*t*_1_–*t*_8_).

The subjects sat comfortably, with their right upper limb resting in a relaxed position on a horizontal support. The support was formed by a polystyrene bead vacuum splint and molded to the hand, palm and forearm of the subject. This device enabled the limb to be loosely restrained to maintain the horizontal alignment of the longitudinal axis of the pronated hand with the forearm and to keep it pointing toward the central vertical meridian (**Figure 1A**). The head was stabilized using a combination chin rest and head support device. Visual stimuli were rear projected on a wide-tangent black screen (160 cm in width and 120 cm in height) that was placed 1 m in front of the subject. The participants had to fixate on a white central cross. After a variable time interval of 2–5 s after a warning tone, a colored square (subtending 0.6° of visual angle) appeared for 2 s at 5° from the central cross along the horizontal meridian, in the left or right visual field. The color of the peripheral stimulus was randomly set to be blue or yellow. The color of the visual stimulus determined whether the subjects had to quickly respond by moving their gaze to the square and fixating it as accurately as possible (overt orienting of visuo-spatial attention; ‘go’ condition), or, conversely, were required to maintain their gaze on the central cross (covert orienting of visuo-spatial attention; ‘no-go’ condition). The turning off of the peripheral stimulus marked the beginning of a new trial.

Single-pulse TMS was randomly delivered within each trial at one of 10 possible time epochs (**Figure 1B**): 500 ms before the warning tone (*b*_1_), 500 ms before the visual stimulus appeared (*b*_2_) or at one of eight different epochs between 120 to 960 ms after the onset of the peripheral cue, corresponding to multiples of 120 ms time delays (*t*_1_ to *t*_8_). At *b*_1_ and *b*_2_, TMS was delivered before the visual stimulus appeared to measure muscle excitability in the resting condition (‘baseline stimuli’), when the subjects were still unable to determine which ocular task they had to execute, which depended on the color of the visual target. At the time delays *t*_1_ to *t*_8_ (‘test stimuli’), TMS was aimed at describing the time course of the excitability changes occurring during the oculomotor task.

The left/right and ‘go’/‘no-go’ stimulus conditions as well as TMS pulses at the different time delays were randomly intermixed within the experimental session so the subjects were completely unable to predict the eye task to perform and the timing of the TMS occurrence. Each experimental session comprised five blocks of 68 trials (with 3 min intervals between blocks), yielding an overall number of 340 trials [10 trials for each of the 2 baseline stimuli plus 10 trials for each of the 32 possible test conditions (2 sides × 2 ‘go’/‘no-go’ conditions × 8 time delays)]. The assignment of ‘go’/‘no-go’ condition to the color of the peripheral stimulus was randomly balanced across subjects.

Particular attention was paid when explaining the task to avoid drawing the subject’s attention to the possibility of making an aiming movement of the arm toward the target. In addition, the lack of any imagery of manual pointing movements was assessed through a subject interview after the experimental session. In order for a trial to be included in the analysis, the following criteria had to be fulfilled: (1) the subject kept his upper limb muscles completely relaxed, as defined by the absence of any detectable EMG activity during the entire trial duration; (2) the ocular behavior was coherent with the response demanded by the color code of the peripheral attentional stimulus, that is, a saccade was executed only when required. For each muscle, the detection of a spontaneous contraction during the task yielded an average exclusion of 1.1% of the trials (range: 0.0–7.9%).

### Eye Movement and EMG Recording

Horizontal and vertical eye movements were recorded (DC 200 Hz low-pass filtered) by means of electrooculography (EOG). Ag-AgCl electrodes were placed at the external canthi of both eyes and above and below the right eye. During an experimental session, EOG calibration was repeated at each interval between trial blocks. Drift of DC offset was compensated within each trial by making the subject look at the central fixation cross before task onset. Surface electromyograms (EMG) were simultaneously recorded on the right-hand side from three distal upper limb muscles: *first dorsal interosseous* (FDI), *abductor digiti minimi* (ADM), and *extensor carpi radialis* (ECR) muscles (1000 × amplification; 0.2 Hz – 2 kHz bandwidth). Besides being easily stimulated by low intensity TMS, these muscles have been recently demonstrated to exhibit direction-specific excitability changes following visually guided saccades (Falciati et al., 2013). Attention was paid to ensure that the subjects kept their muscles completely relaxed, as demonstrated by the absence of any detectable EMG activity for the entire duration of the task.

The EOG and EMG signals were digitally converted at a sampling rate of 5 kHz (National Instruments PCI-MIO-16E-4) and analyzed oﬄine by means of custom LabVIEW (National Instruments, Austin, TX, USA) software. Saccade onset was measured by identifying the peak of eye acceleration at the beginning of the ocular response.

### Transcranial Magnetic Stimulation

A 70 mm figure-eight double coil connected to a MagStim Super Rapid magnetic stimulator (Mag-1450-00, MagStim Co. Ltd Whitland, UK) was positioned over the left motor cortex, contralateral to the EMG-recorded muscles. TMS procedures complied with the general guidelines proposed by International Federation of Clinical Neurophysiology (Rossini et al., 2015). The coil was placed tangentially to the scalp, with the handle pointing backward and laterally at a 45° angle to the sagittal plane. This orientation was chosen because the lowest motor threshold is achieved when the induced electrical current in the brain flows approximately perpendicularly to the central sulcus (Mills and Nithi, 1997). The scalp site at which MEPs were elicited in the FDI muscle at the lowest stimulus strength was determined. Once the optimal scalp site was found, the coil was securely fixed in place by means of an appropriate mechanical device. The response threshold was defined as the stimulus intensity at which 5 out of 10 consecutive single TMS pulses at the optimal site evoked an MEP with an amplitude of at least 100 μV in the relaxed muscle. During the entire stimulation paradigm, stimulus intensity was set at 1.2 times the FDI motor threshold. The mean stimulation intensity across subjects was equal to 67.9% of the maximum power of the magnetic stimulator. At the optimal scalp site for FDI, this stimulation intensity also evoked MEPs in the ADM and ECR muscles in almost all of the experimental sessions, although generally with a considerably lower amplitude. However, to ensure that the excitability changes were measured against a reliable baseline, the subjects were included in the analysis only if, within each muscle, the MEPs obtained when TMS pulses were delivered during the control phase (‘baseline stimuli’) of the experimental protocol (i.e., before the peripheral visual stimulus onset) had a mean amplitude greater than 50 μV. This acceptance criterion was fulfilled in 18 and 17 subjects for the ADM and ECR muscles, respectively. Furthermore, 2 out of the 20 subjects were excluded from analysis of the FDI muscle due to technical recording issues.

### Data Processing and Statistical Analysis

The peak-to-peak amplitudes of the MEPs recorded from the investigated muscles were measured trial by trial. For each subject, a paired-sample *t*-test was applied separately for the FDI, ADM, and ECR muscles to compare the mean MEP amplitudes obtained at the TMS delays *b*_1_ and *b*_2_, i.e., during fixation of the central cross prior to the warning tone and the visual stimulus, respectively. Because none of the comparisons yielded a statistically significant difference between the two measures (*P* > 0.07), in every subject, a single control value was computed for each muscle by averaging the *b*_1_ and *b*_2_ responses (MEP_baseline_). This baseline value was then used to normalize the raw MEP amplitudes recorded in each muscle. Mean normalized MEP amplitudes were computed within each subject for all trials of a given stimulation condition (target side and time binning), and used for subsequent statistical analyses.

Because one of the main aims of this study was to ascertain whether the observed changes in muscle excitability were triggered by the visual stimulus presentation or were time locked to eye movement execution, statistical analysis of the ‘go’ trials was performed on the mean MEP amplitudes computed after the responses were aligned with either target onset (*stimulus-locked analysis*) or the beginning of the eye movement (*saccade-locked analysis*).

**FIGURE 2:**
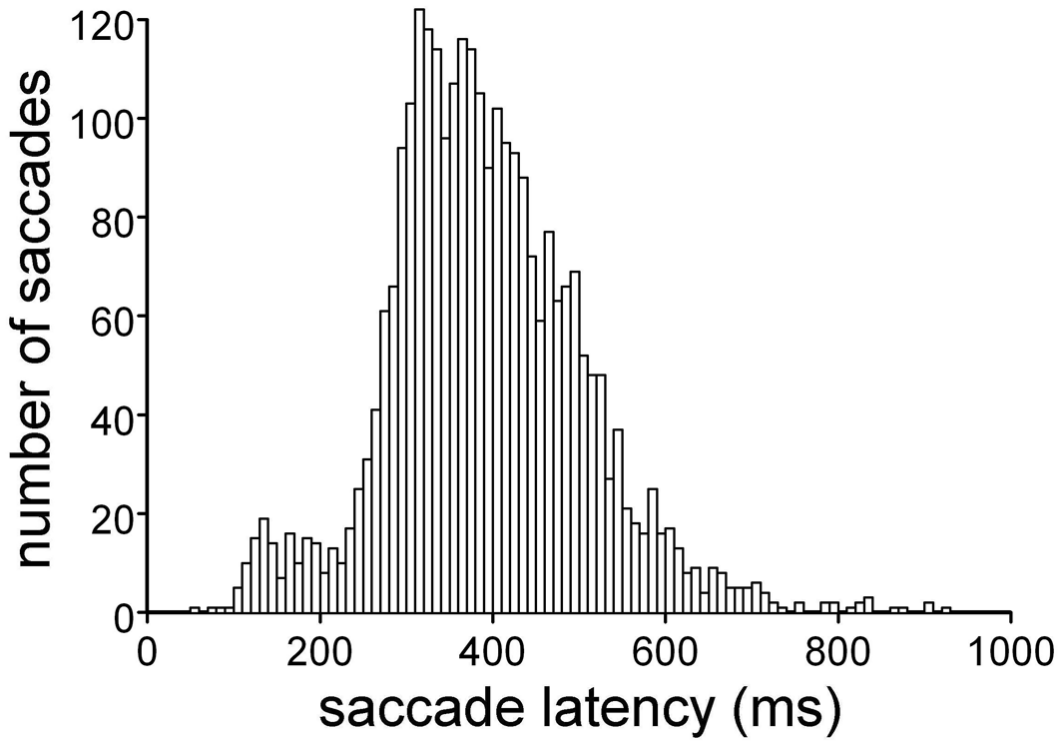
**Distribution of saccade latencies in the ‘go’ trials.** Execution of the double-choice discrimination task affects the ocular responses, which show long latencies and large variability.

The stimulus-locked analysis was performed by simply computing the average amplitude of the MEPs recorded at the different TMS time delays, *t*_1_ to *t*_8_. By contrast, the saccade-locked analysis was performed by exploiting the very large statistical variability of the latency of the eye movement response present in this double-choice task (**Figure 2**). By computing TMS latency with respect to the start of the eye movement, binning of the transformed TMS delays was performed by pooling all trials that fell within a given time interval with respect to saccade onset. To be consistent with the discretization of the stimulus-locked analysis, for every muscle, trials were grouped and averaged within each subject, using equally spaced, 120 ms bins of TMS delays, the central values of which fell at -240, -120, 0, 120, 240, 360, 480, and 600 ms with respect to saccade onset. Therefore, the mean MEP amplitude of each bin was computed by averaging the responses elicited by TMS pulses occurring in a ± 60 ms time interval around the central bin value.

To ensure a minimum number of observations, only bins with three or more MEPs were included in the analysis. As a result of this procedure, an average of 8.0 ± 2.5 trials per bin was pooled in all of the muscles.

## Results

### Saccadic Latencies

The subjects were taught to discriminate the color of a peripheral attentional stimulus, which appeared while they were fixating a central cross. According to a color code, the subjects were instructed to execute a saccade toward the peripheral stimulus (‘go’ condition) or to maintain their gaze on the central fixation cross (‘no-go’ condition). **Figure 2** shows the distribution of the saccade latencies obtained for the ‘go’ condition. This double-choice task, in which the decision to make an eye movement must be made depending on the color of the target, results in quite long response latencies, with large statistical variability within each subject. The average saccade latency across the participants was 391 ± 113 ms. Nevertheless, all subjects performed the task with high accuracy, as only an average of 6.7% of the trials were rejected for errors in task execution (range: 0.9–14.0%).

The large variability in saccade latency was exploited to discriminate between stimulus-linked and saccade-linked modulation of MEP amplitude, by aligning the timing of the TMS responses either with presentation of the visual stimulus or with saccade onset.

### Stimulus-locked Changes in CSS Excitability

**FIGURE 3:**
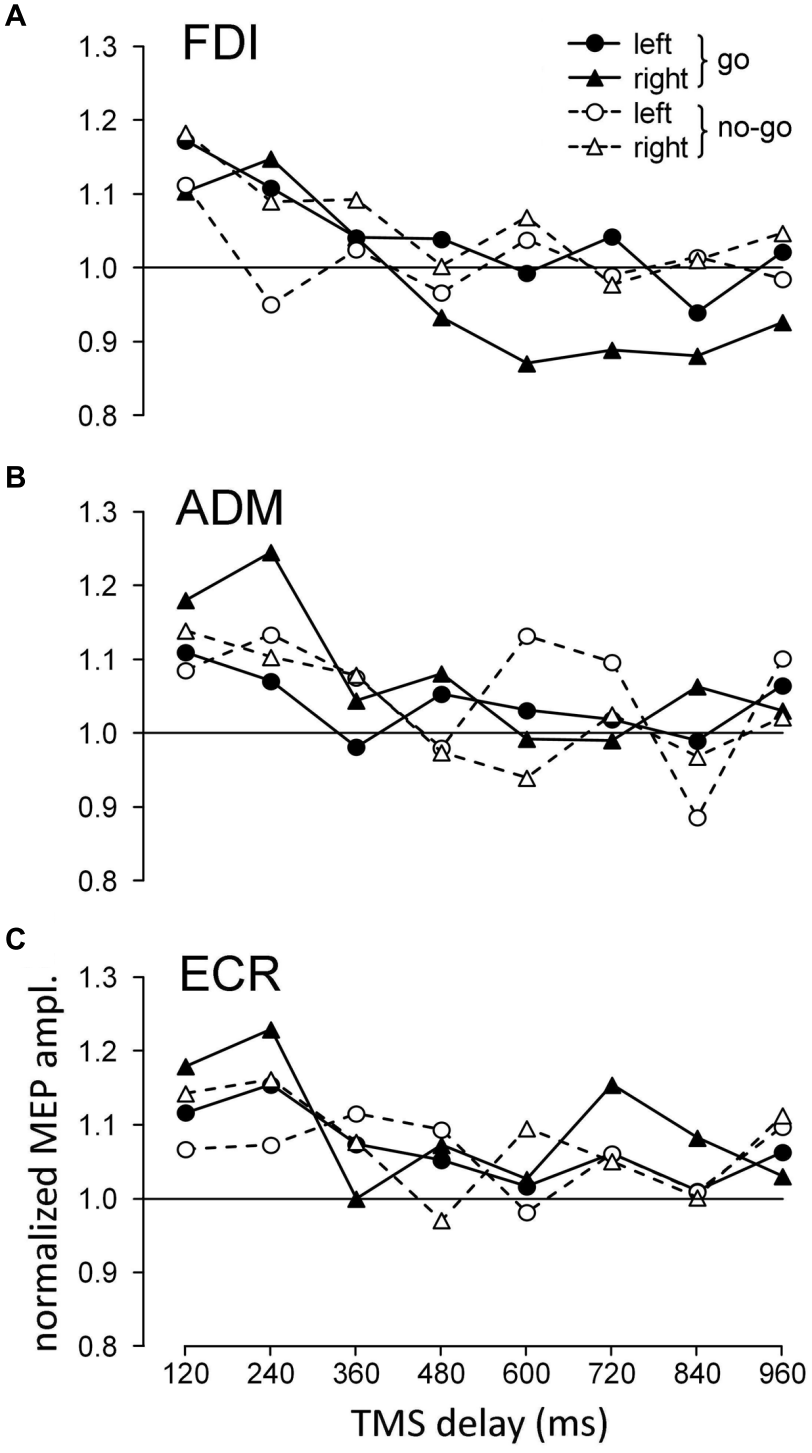
**Graphs depicting the time course of the changes in the mean normalized motor evoked potential (MEP) amplitudes with respect to stimulus onset, recorded from the first dorsal interosseous (FDI) **(A)**, ADM **(B)**, and ECR **(C)** muscles as a function of cue side and ocular task (‘go’/‘no-go’).** Values higher than unity indicate MEP amplitudes larger than baseline.

At different time delays from the onset of the attentional stimulus, a single pulse of TMS was delivered to the left motor cortex, and MEPs were recorded from the contralateral relaxed upper limb. **Figure 3** shows the average change in MEP amplitude for each recorded muscle across all subjects as a function of TMS delay and the visual hemifield in which the attention target appeared. The data were normalized within each subject by the mean MEP amplitude of the trials in which TMS occurred before presentation of the visual stimulus (baseline).

To test the statistical significance of the illustrated changes in CSS excitability, three-way repeated measures ANOVA (**Table 1**) was performed on the mean normalized MEP amplitudes of each subject for each muscle, with ‘*task*’ (‘go’ vs. ‘no-go’ task condition), ‘*side*’ (presentation of the visual stimulus in the left vs. right hemifield) and ‘*TMS delay*’ (120, 240, 360, 480, 600, 720, 840, and 960 ms after target onset) as grouping factors. The analysis demonstrated that in all muscles, ‘*TMS delay*’ was the only significant principal effect. Furthermore, a significant ‘*task* × *side*’ interaction was also present for the FDI muscle. No other statistically significant interactions among the factors were found.

**Table 1:** Three-way repeated measures ANOVA of stimulus-locked motor evoked potential (MEP) amplitudes.

	FDI (*N* = 18)		ADM (*N* = 18)		ECR (*N* = 17)	
	*F_df,(*N*-1)x31_*	*P*	*F_df,(*N*-1)x31_*	*P*	*F_df,(*N*-1)x31_*	*P*
task	1.225	0.270	0.255	0.614	0.445	0.505
side	0.231	0.631	0.028	0.868	1.151	0.284
TMS delay	**3.727**	0.001**	**2.290**	0.026**	**2.590**	0.012**
task × side	**6.893**	0.009**	1.792	0.181	0.126	0.723
task × TMS delay	1.184	0.310	0.745	0.634	0.838	0.556
side × TMS delay	0.678	0.691	0.959	0.460	0.919	0.491
task × side × TMS delay	0.098	0.998	0.252	0.971	0.575	0.777

The degrees of freedom (*df*) for ‘task’, ‘side’, and ‘task × side’ were equal to 1, and the *df* for ‘TMS delay’ and corresponding interactions were equal to 7. Bold value and ** indicates statistical significance (*P* < 0.05).

Simple inspection of **Figure 3** suggests that the principal effect of ‘*TMS delay*’ was generally caused by the fact that MEP amplitude was considerably larger in all muscles soon after the onset of the attention target compared to later on in the trial. By contrast, interpretation of the significant ‘*task* × *side*’ interaction for the FDI muscle requires more careful analysis.

In the ‘go’ condition, the MEPs showed similar amplitudes for both sides of visual stimulation at short TMS delays but became clearly different in size depending on target position for TMS latencies larger than 360 ms. In fact, while MEP amplitudes for the left visual stimuli returned to baseline, similar to the responses in the ‘no-go’ condition, ‘go’ trials with visual stimuli on the right (ipsilateral) side yielded clear inhibition of FDI muscle excitability with respect to the control.

To statistically confirm these changes in FDI muscle excitability, two-way repeated measures ANOVA with ‘*side*’ and ‘*TMS delay*’ as grouping factors was performed, separately for the ‘go’ and ‘no-go’ tasks, on the mean normalized amplitude of MEPs obtained at TMS delays of 480–960 ms. Only the principal effect of ‘*side*’ in the ‘go’ condition reached statistical significance [*F*_(1,153)_ = 9.235, *P* = 0.003]; on average, MEPs were smaller in the presence of a right/‘go’ target. Furthermore, no significant difference in MEP amplitude (two-way ANOVA with ‘*TMS delay’* and ‘*task’* condition as factors) was found at the corresponding TMS delays among the responses recorded in the ‘no-go’ and left/‘go’ trials. These findings demonstrate that approximately 500 ms after the onset of a peripheral target, significant inhibition is induced in the FDI muscle when the task requires that an eye movement be made to the ipsilateral side. By contrast, in the ‘no-go’ trials, no changes in FDI excitability were present within the same time epoch as a function of the spatial location of the cue. This differential modulation of FDI excitability between ‘go’ and ‘no-go’ trials, which depended on the side on which the target of attention was presented, can account for the significant ‘*task* × *side*’ interaction found in the three-way ANOVA described above. Finally, inspection of the data for the ‘no-go’ condition also suggests that the mean MEP amplitude in the FDI muscle at a TMS delay of 240 ms was smaller with contralateral with respect to ipsilateral visual stimuli. However, this difference was not statistically significant (paired *t*-test, *P* = 0.560).

**FIGURE 4:**
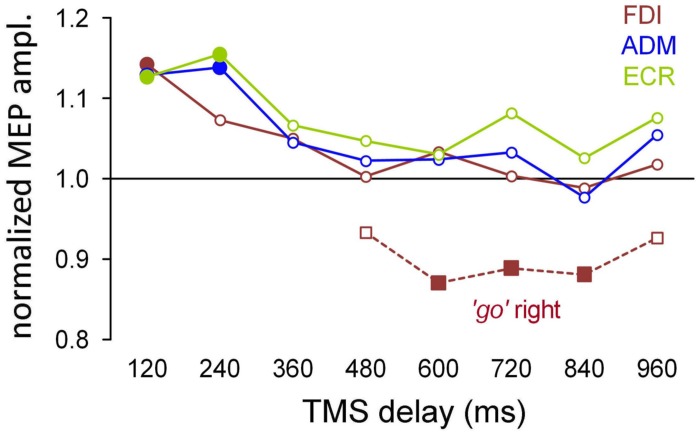
**Mean MEP amplitudes across subjects as a function of TMS delay with respect to stimulus onset.** The data values for the ADM and ECR muscles were averaged irrespective of the ocular task and cue side. By contrast, MEP amplitudes of the FDI muscle from the ‘go’ trials with targets on the right side were averaged separately (squares) because they were significantly smaller than in the other experimental conditions (see text). Filled symbols denote a statistically significant difference from the baseline (one-sample *t*-test; *P* < 0.05). Values higher than unity indicate MEP amplitudes larger than baseline.

**Figure 4** summarizes the mean excitability changes across subjects in each of the recorded muscles as a function of TMS delay with respect to the onset of the attentional visual stimulus. The data points were computed by pooling experimental conditions that did not yield statistically significant differences in the ANOVAs described above. Thus, the graph values for the ADM and ECR muscles are the grand means of the normalized MEP amplitudes, irrespective of the ocular task and the side of the visual stimulus. By contrast, right/‘go’ trial responses were excluded from the averaging procedure for the FDI muscle and were plotted separately (red squares) in **Figure 4**, as their MEP amplitudes have been demonstrated to be statistically smaller than in the other experimental conditions. Furthermore, filled symbols denote a statistically significant difference between the baseline and the corresponding mean MEP amplitude (one-sample *t*-test; *P* < 0.05).

It is interesting to observe that after exclusion of the right/‘go’ trials in the FDI averaging procedure, there was a very tight overlap of the time courses of the changes in CSS excitability for all three of the recorded muscles. That is, in both the ‘go’ and ‘no-go’ tasks, the presentation of a task-relevant visual stimulus induced a generalized increase in MEP amplitude of approximately 15% within the first 250 ms, followed by a gradual return to baseline within approximately 400 ms after the onset of the peripheral visual stimulus. On top of this generalized modulation of upper limb CSS excitability, the FDI muscle exhibited side-specific modulation in the ‘go’ condition. That is, prolonged muscle inhibition develops at TMS time delays longer than 500 ms when the oculomotor response has to be made to the ipsilateral side. Notice that this inhibition appears to follow execution of the eye movement because the mean saccade latency in the ‘go’ trials was 391 ms.

### Saccade-locked Changes in CSS Excitability

**FIGURE 5:**
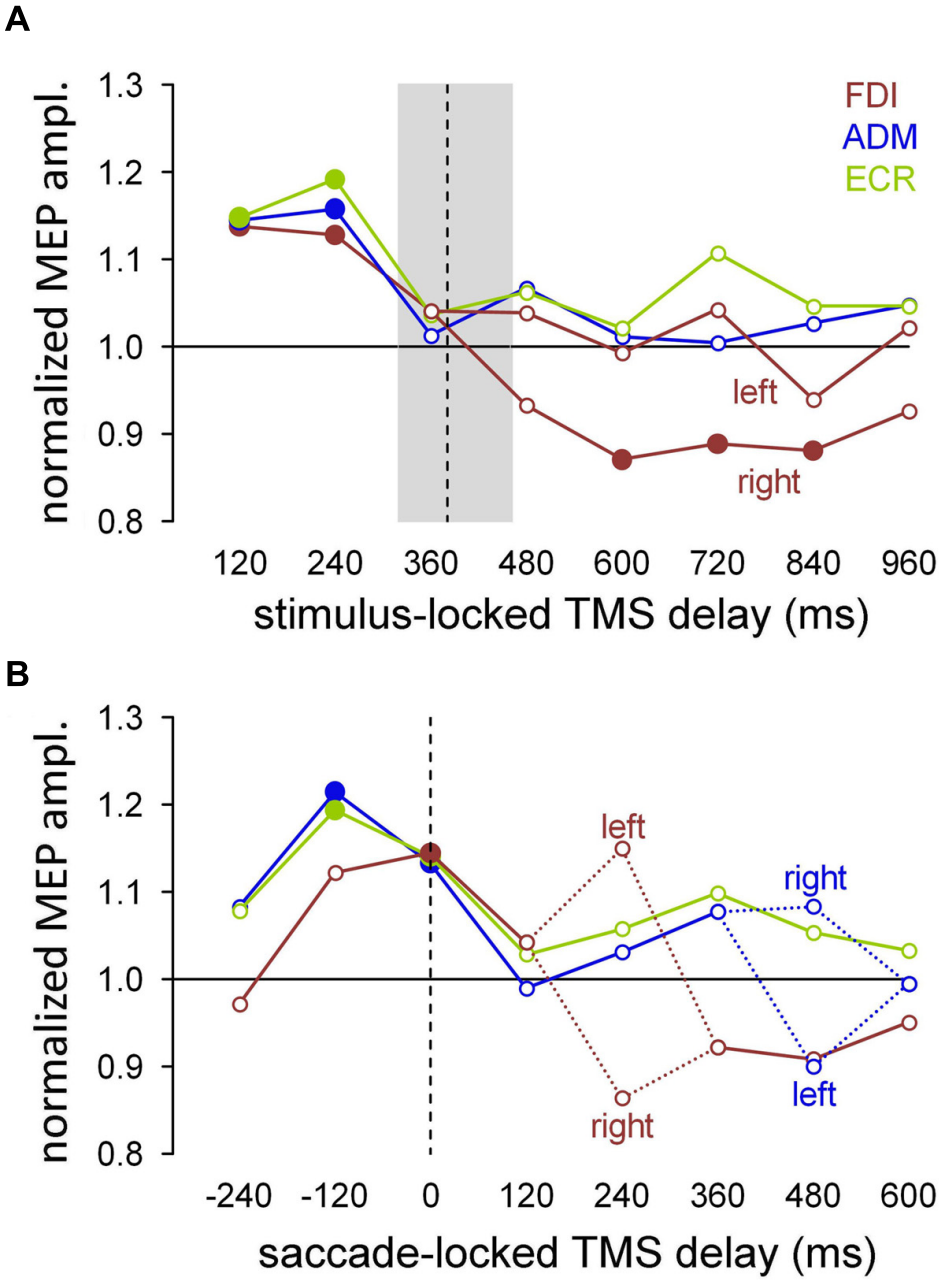
**For each muscle, modulation of the mean amplitude of MEPs recorded during the ‘go’ trials.** Filled symbols denote a statistically significant difference from the baseline (one-sample *t*-test; *P* < 0.05). **(A)** Stimulus-locked analysis performed by averaging the MEP amplitudes for each TMS delay with respect to the visual stimulus. In the FDI muscle, the responses were separately averaged as a function of the target side for TMS delays longer than 360 ms. The median value (vertical dashed line) and the interquartile range (gray shade) of the saccade latencies are also shown. **(B)** Saccade-locked analysis performed by aligning the occurrence of TMS to saccade onset. Mean MEP amplitudes were computed after grouping the TMS delays with respect to the beginning of the eye response, in equally spaced bins of 120 ms. Figures drawn along the *X*-axis represent the bin central values used for the averaging procedure. Negative values indicate that the MEPs were elicited before the ocular response. Dashed lines connect the mean values for the left and right visual stimuli, for the bins that yielded a statistically significant difference in MEP amplitude relative to the side of target appearance. *Y*-axis values higher than unity indicate MEP amplitudes larger than baseline.

The saccade-locked analysis was performed by binning the TMS responses by grouping all trials that fell within a given time interval with respect to execution of the eye movement for each muscle. The mean MEP amplitudes were computed within each subject for equally spaced bins of ±60 ms, embracing a TMS time interval between -300 and 660 ms around saccade onset. **Figure 5** shows the grand means of the normalized MEP amplitudes, comparing the values obtained with the stimulus-locked and saccade-locked analysis of the TMS responses recorded in ‘go’ trials, that is, when the subjects performed an explicit saccadic eye movement toward the attentional stimulus.

To make the comparison on the same pool of data, stimulus-locked MEP amplitudes (**Figure 5A**) were recomputed only for the ‘go’ trials. In agreement with the previously described statistical analysis, FDI MEPs recorded at TMS delays between 480 and 960 ms after stimulus onset were averaged separately depending on the side of the visual target presentation. The median value (vertical dashed line) and the interquartile range (gray box) of the saccade latencies are also depicted, to evaluate the timing of the muscle excitability changes with respect to the oculomotor response. As shown in **Figure 4**, CSS excitability increased by approximately 15% in all tested muscles immediately after stimulus presentation, then decreased to the baseline level around saccade onset. In agreement with the previous analysis, the excitability of the FDI muscle significantly decreased below baseline following a saccade to the right (ipsilateral) side. As usual, the filled symbols indicate a statistically significant difference with respect to the baseline (one-sample *t*-tests, *P* < 0.05).

When the MEP analysis was performed by aligning the occurrence of TMS with respect to saccade onset (**Figure 5B**), the data could be interpreted in a new perspective. In fact, the changes in MEP amplitude show components that are highly correlated with the timing of the oculomotor response. Specifically, the results show an increase in the overall CSS excitability building up shortly before the start of the eye movement as well as some side-dependent modulation in MEP amplitude occurring at specific narrow time epochs after saccade execution.

To test the presence of a statistically significant effect of the target side on CSS excitability, repeated measures ANOVA was performed on the mean MEP amplitudes of each muscle for each saccade-locked time bin, with ‘*saccade direction*’ (leftward vs. rightward) as the grouping factor. In the absence of a statistically significant difference, mean MEP amplitudes were computed irrespective of stimulation side.

The graph in **Figure 5B** depicts the presence of a generalized saccade-locked buildup of excitability, starting in all muscles at approximately 200 ms before the onset of the ocular response and returning to the baseline level shortly after the end of the eye movement. Specifically, at the time bin centered around -120 ms, MEP amplitude was significantly larger (filled symbols) than baseline in the ADM and ECR muscles [*t*_(16)_ = 2.407, *P* = 0.014; *t*_(15)_ = 2.833, *P* = 0.006, respectively]. By contrast, at the 0 ms time bin, all three of the muscles showed a significant increase in mean MEP amplitude [FDI: *t*_(16)_ = 2.427, *P* = 0.014; ADM: *t*_(16)_ = 2.008, *P* = 0.031; ECR: *t*_(15)_ = 1.879, *P* = 0.040]. Interestingly, in all muscles, MEP amplitude did not differ from baseline in the time bin centered at 240 ms before saccade onset, confirming that the timing of this excitability modulation was strictly bounded to the oculomotor response.

By contrast, direction-specific modulation of MEPs was found in the FDI and ADM muscles only at particular TMS delays. At the time bin centered at 240 ms (**Figure 5B**, red dotted lines), the mean MEP amplitude in the FDI muscle was larger after leftward saccades than after rightward saccades [*F*_(1,17)_ = 8.303, *P* = 0.010]. Conversely, at the 480 ms time bin (**Figure 5B**, blue dotted lines) the opposite excitability change occurred in the ADM muscle, as MEP amplitude was significantly larger after rightward saccades than after leftward saccades [*F*_(1,17)_ = 5.955, *P* = 0.026]. No statistically significant side-dependent modulation in excitability emerged for the ECR muscle.

The presence in the FDI saccade-locked analysis of a precisely timed, wide difference in MEP amplitude that depended on the side of target occurrence also shed new light on the stimulus-locked MEP analysis for that muscle. In fact, the long-lasting decrease in excitability observed in the ‘go’ trials, when the visual target was presented on the right side, may very well be interpreted as the spreading in time of the saccade-locked decrease in MEP amplitude, which occurs in a narrow temporal window at 240 ms after the onset of the eye movement. This interpretation is supported by the very large variability of saccade latency in our double-choice task (**Figure 2**), which is expected to introduce a spread of at least 400 ms when a saccade-locked, short-lasting event is represented in a stimulus-locked time scale.

The saccade-locked analysis demonstrated that the oculomotor response determines a generalized increase in CSS excitability, starting at approximately 200 ms before saccade onset. However, an excitability increase was also present in all muscles in the ‘no-go’ trials, even if an eye response was actively inhibited. Furthermore, the amplitude of MEPs recorded shortly after the peripheral cue presentation was not significantly different between ‘go’ and ‘no-go’ trials, suggesting that the generalized increase in CSS excitability observed before the ocular response was also related, at least partially, to the presentation of the attentional stimulus.

To ascertain whether the shift of visual attention contributed to the increase in overall muscle excitability in the ‘go’ trials as well, we analyzed the changes in MEP amplitude induced by a TMS pulse delivered at 120 ms after the attentional stimulus (first test stimulus *t*_1_) when the saccade latency was longer than 300 ms. In this case, the oculomotor response occurs too late to affect CSS excitability at this early TMS time delay (see **Figure 5B**) and, therefore, the possible changes in MEP amplitude must necessarily be ascribed to sensory processing of the visual stimulus, rather than to motor programming.

**FIGURE 6:**
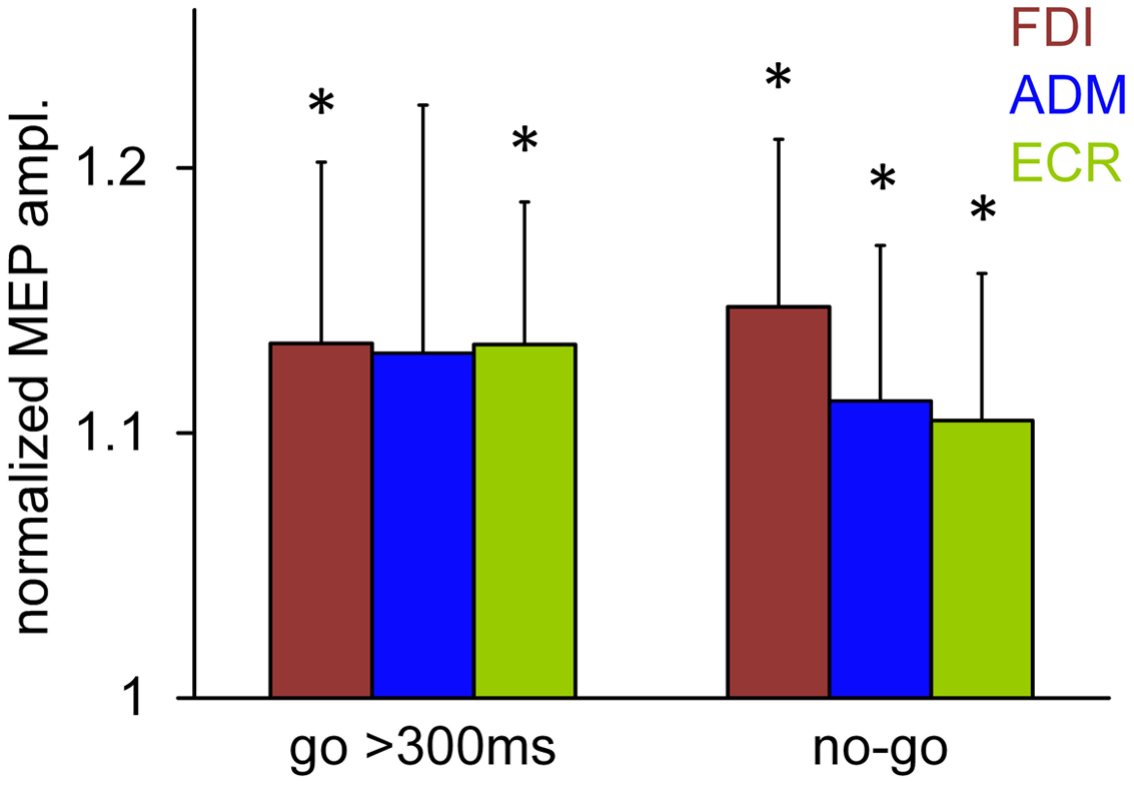
**Normalized mean MEP amplitudes recorded in the ‘go’ trials with a TMS delay of 120 ms and a saccade latency greater than 300 ms, compared to the responses observed in the ‘no-go’ trials recorded at the same TMS delay after stimulus presentation.** Bars represent the standard error of the mean. Asterisks indicate that the values are significantly greater than baseline (one-sample *t*-test; *P* < 0.05).

**Figure 6** compares the normalized mean MEP amplitudes recorded in the ‘go’ trials with a TMS delay of 120 ms and saccade latencies greater than 300 ms with the corresponding mean values observed in the ‘no-go’ trials recorded at the same TMS delay after the presentation of the visual stimulus. Asterisks indicate statistical significance. It is evident that the mean MEP amplitudes from ‘go’ trials are approximately 13% greater than baseline, even in the absence of the facilitatory effect that precedes the execution of a saccadic eye movement. However, while the MEP amplitudes from the FDI and ECR muscles were found to be significantly greater than baseline (one-sample *t*-test; *P* = 0.033 and 0.012, respectively), the increase in the ADM value did not reach statistical significance (*P* = 0.091). Furthermore, it should be noted that this increase in MEP amplitude that was recorded in the ‘go’ trials with a late saccadic response did not differ from that found in the ‘no-go’ trials at the same TMS delay (120 ms). In fact, two-way repeated measures ANOVA performed on the mean values shown in **Figure 6**, with ‘*muscle’* and ‘*condition’* (go/no-go) as factors, yielded no statistically significant principal effects or interactions. This finding demonstrates that in the ‘go’ trials as well, the non-spatially coded increase in muscle excitability preceding saccade onset had a component that was time locked with the presentation of the visual stimulus.

## Discussion

We have found that when subjects perform a task in which a decision to make a gaze movement is based on discrimination of the visual properties of a target, a covert motor plan of the hand is produced even if a manual response is not required but only in the trials in which a saccadic eye movement is actually executed. The saccade-locked time analysis of MEP amplitudes revealed a direction-specific modulation of excitability in the FDI and ADM muscles at narrow time intervals of 240 and 480 ms, respectively, after saccade onset. By contrast, if the time course of the changes in MEP amplitude were analyzed with respect to the occurrence of the visual stimulus, only a non-specific increase in excitability was observed in all recorded muscles, which was unrelated to the side of occurrence of the target. In fact, stimulus-locked excitability changes in ‘go’ trials are undistinguishable from the transient generalized increase in muscle excitability which is observed following a covert shift of attention in ‘no-go’ trials.

The direction-specific MEP modulation following the gaze movement is compatible with a coarse sub-threshold motor program encoding a finger movement in the direction of the preceding saccade. In fact, the FDI muscle on the right-hand side, the activation of which during a pronated hand posture produces a leftward deviation of the index finger, showed higher MEP amplitudes after leftward compared to rightward saccades. Conversely, the excitability changes in the ADM muscle, the activation of which induces a rightward deviation of the little finger, were opposite to those observed in the FDI muscle, i.e., MEP amplitudes were largest following rightward saccades.

These data are consistent with the results of a previous paper (Falciati et al., 2013), in which direction-specific changes in upper limb CSS excitability, compatible with a covert motor plan of aiming the hand at the same target of gaze, were described at approximately 150 ms after the onset of visually guided saccades, in the absence of any manual response. However, those findings were based on a speeded reaction time task, yielding an extremely small variability of saccade latency. This fact makes it impossible to ascertain whether MEP modulations are temporally linked to the visual stimulus or to the eye movement. By contrast, the ‘go’/‘no-go’ color discrimination task employed in the present experimental protocol revealed a more than threefold increase in reaction time variability. Interestingly, direction-specific and forearm posture-related changes in the CSS excitability of the relaxed upper limb have also been described during smooth pursuit eye movements (Maioli et al., 2007).

The results of this paper are relevant within the context of some influential models about the relationship between visual attention and action representation in ‘pragmatic maps.’ According to the ‘Premotor Theory’ of attention (Rizzolatti et al., 1994), the attentive selection of a spatial location is the result of the activation of motor programs for possible goal-directed movements toward it. By contrast, the “Visual Attention Model” (Schneider and Deubel, 2002) postulates a common attention mechanism that induces the activation of multiple effector motor programs toward the target object, independently of the intention to make a real movement. However, while a tight overlap between oculomotor and spatial attention maps has indeed been widely demonstrated by human neuroimaging (e.g., Corbetta et al., 1998; Nobre et al., 2000) and monkey neurophysiology studies (e.g., Schall et al., 1995; Moore and Fallah, 2001), experimental evidence regarding the idea that the attentive selection of a spatial location is associated with the planning of a manual aiming movement is very limited and indirect.

This study fails to support the predictions of both these attention models, at least when they are generalized to include spatially coded limb movements. In our experimental paradigm, attentive discrimination of the target color is required in both ‘go’ and ‘no-go’ trials to determine the response strategy. If the orientation of spatial attention were tightly linked to the activation of multiple motor maps (notably manual and ocular), we would expect to observe direction-specific MEP modulations time locked to the presentation of the visual stimulus in both experimental conditions. Conversely, a change in CSS excitability compatible with a motor plan encoding a manual aiming movement was found to occur only after the end of an overt gaze movement in the ‘go’ trials, with very precise timing with respect to saccade onset. This result strongly supports the view of a strict coupling between the planning of a manual response and overt gaze orienting (Land et al., 1999; Maioli et al., 2007; Mennie et al., 2007; Falciati et al., 2013).

As a corollary, it is worth to point out that a covert planning for a manual movement is bound to the actual execution of an eye movement, and not to saccade programming. This distinction arises in light of the widely accepted theory that a covert orienting of spatial attention is always linked to the preparation of a saccade program (Hoffman and Subramaniam, 1995; Deubel and Schneider, 1996; Schneider and Deubel, 2002; Baldauf and Deubel, 2008). Since covert orienting of attention in ‘no-go’ trials does not elicit changes in CSS excitability, we have to conclude that forced coupling of manual and eye motor programs is not a built-in property of the control system of visually guided motor behavior. This is at odds with what is predicated by some eye-hand coordination models in which separate effector-specific controllers are driven by a common attentional modulation or a common command input (cfr Dean et al., 2011). By contrast, in our saccade task, a covert hand movement program was activated only at the end of a top-down decision-making process regarding the actual execution of an eye movement, based on the color discrimination of the visual stimulus.

These findings suggest that the execution of a spatially directed motor response, regardless of whether it is exogenously or top-down driven, automatically facilitates a covert eye-hand coordination program, which is normally scheduled to achieve the most appropriate goal-directed motor action. Indeed, in common natural tasks, eye and arm movements are tightly linked, making manual aiming more accurate compared with when the hand moves alone (Vercher et al., 1994; Henriques et al., 1998; Neggers and Bekkering, 2000, 2002; van Donkelaar and Staub, 2000; Medendorp and Crawford, 2002; Horstmann and Hoffmann, 2005). The eyes begin to move between 70 and 90 ms before the arm, and the hand arrives at the target in less than 500 ms after the beginning of the eye movement (Carnahan and Marteniuk, 1994; Lünenburger et al., 2000; Sailer et al., 2000; Dean et al., 2011). It should be noted that in an eye-hand coordination task, the timing of the manual movement relatively to the gaze shift closely corresponds to the occurrence of the direction-specific modulation of MEP amplitude, which was observed in the purely oculomotor task of this study.

In addition to the described spatially coded changes in MEP amplitude, the data analysis has also demonstrated the occurrence of a generalized increase in CSS excitability in the ‘go’ trials before the onset of the eye response. Similar modulation of MEP amplitude was also observed in the ‘no-go’ trials after the presentation of the visual stimulus. In the ‘go’ trials, this increase in muscle excitability has been demonstrated to result from the sum of at least two distinct components. The first component appears to be time locked to the sudden appearance of the peripheral cue. The second one consists of a buildup of excitability starting approximately 150 ms before saccade onset and therefore, is time locked to eye movement execution. These data clearly show that, indeed, the allocation of visual attention to a spatial location activates the motor map of the arm. However, the lack of muscle and direction specificity argues against the possibility that this activation is compatible with attentional selection for the preparation of a manual movement (Schiegg et al., 2003) or with a space representation within the hand ‘pragmatic map’ (Rizzolatti et al., 1994; Schneider and Deubel, 2002). Furthermore, the buildup of CSS excitability preceding the eye response is reminiscent of the increase of activity described in the frontal eye fields and superior colliculus during the decision-making process for saccade initiation (Hanes and Schall, 1996; Sparks et al., 2000; Brown et al., 2008; Schall et al., 2011; Jantz et al., 2013). However, the view that this pre-saccadic buildup is related to a decisional process for the execution of a manual response is also unlikely. In fact, muscle excitability reaches its highest peak well before the start of the eye response and decays to control levels at 120 ms after saccade onset, that is, before the start of any direction-specific modulation of MEP amplitude in the FDI and ADM muscles.

A possible interpretation of these findings is that the attentional selection of a space location and the decision-making processes about the choice of the appropriate oculomotor response determine an overall excitability increase in the hand motor map. This facilitation could be finalized at the enhancement of the readiness of the limb control system to execute a possible goal-directed action involving eye-hand coordination, as this is one of the most likely behaviors in common spatially oriented motor tasks. Once a decision about the appropriate oculomotor strategy is made (that is, suppression of the saccadic program to maintain gaze on the central fixation point or the execution of an eye movement toward the peripheral cue), this generalized facilitation is turned off because a manual response toward the new gaze position is not required in this particular context. It should be noticed how precisely the decay in CSS excitability is centered on the eye movement onset in all recorded muscles. Furthermore, in our experimental task, the covert hand motor plan appeared to be tightly constrained to follow saccade execution and to possibly be part of a unique, serial multi-effector motor program.

## Conflict of Interest Statement

The authors declare that the research was conducted in the absence of any commercial or financial relationships that could be construed as a potential conflict of interest.
